# Four vs. Six Implant Full-Arch Restorations—A Direct Comparative Retrospective Analysis in a Large Controlled Treatment Cohort

**DOI:** 10.3390/jcm14124237

**Published:** 2025-06-14

**Authors:** João Manuel Mendez Caramês, Helena Cristina Oliveira Francisco, Filipe Araújo Vieira, Gonçalo Bártolo Caramês, Jorge Nuno do Rosário Martins, Duarte Nuno da Silva Marques

**Affiliations:** 1 Instituto de Implantologia, Avenida Columbano Bordalo Pinheiro, No. 50, 1070-064 Lisbon, Portugal; helenafrancisco@campus.ul.pt (H.C.O.F.); caramesgoncalo@gmail.com (G.B.C.); jnr_martins@yahoo.com.br (J.N.d.R.M.); duarte.marques@campus.ul.pt (D.N.d.S.M.); 2 Faculdade de Medicina Dentária, Universidade de Lisboa, 1600-277 Lisbon, Portugal; 3 LIBPhys-FCT UIDB/04559/2020, Faculdade de Medicina Dentária, Universidade de Lisboa, 1600-277 Lisbon, Portugal

**Keywords:** edentulous, mandible, maxilla, dental prosthesis, implant-supported, dental prosthesis, arch-supported, atrophy, retrospective study, dental implantation

## Abstract

**Background:** The potential impact of the number of implants (four vs. six) on the implant survival of implant-supported fixed complete dentures (IFCDs) remains inconclusive and subject to ongoing debate. This study compared the implant survival of immediately loaded four vs. six IFCDs, delivered using a patient-centered systematic treatment plan, in a large patient cohort assessed with consistent diagnostic methodology and comprehensive longitudinal follow-up. **Methods:** This retrospective study included records of 943 patients receiving 5989 implants with an average follow-up of 5.0 ± 3.2 (range 0–17) years delivered using a systematic clinical decision support system (CDSS) based on a defined alveolar atrophy classification (CC). Implant survival was analyzed considering predictor variables comprising treatment and anatomic and systemic health-related factors at the overall, maxillary, and mandibular levels using Kaplan–Meier analysis, log-rank tests, and single-predictor and multilevel Cox proportional hazard analysis derived from causal direct acyclic graph methodologies. **Results:** The 2- and 5-year cumulative survival rates (CSRs) of four and six IFCDs were comparable (2-year: 98.6% vs. 98.4%, *p* = 0.362; 5-year: 98.8%, 98.7% *p* = 0.346). The differences between four and six IFCDs were more pronounced at the maxillary levels, specifically after 2 years (97.7% vs. 98.6% *p* = 0.084), and at the mandibular level after 5 years (98.6% vs. 99.4%, *p* = 0.136.). Multilevel Cox proportional hazard models at overall and jaw levels adjusted for confounding factors indicated that implant loss was correlated to jaw type and age at overall and age alone at the mandibular level. Alveolar atrophy (CC) defined within the adopted CDSS was not robustly associated with implant loss. **Conclusions:** Under the guidance of a systematically applied patient-centered CDSS, four and six IFCDs demonstrated high and comparable mid-to-long-term implant survival rates irrespective of the level of analysis or statistical model used to adjust for confounding factors. Prosthetic and technical complications were not evaluated and were, therefore, beyond the scope of this study.

## 1. Introduction

Implant-supported fixed complete dentures (IFCDs) represent a well-established and predictable approach for treating edentulous patients [1,2,3,4,5,6,7,8]. When anatomic, systemic, surgical, and prosthodontic factors are accounted for, the survival rates of immediately loaded IFCDs are comparable to those of early or conventionally loaded equivalents while potentially offering superior patient satisfaction [9,10,11,12].

Although IFCDs have demonstrated significant success, the optimal number and distribution of implants to support them remain subject to ongoing debate. Contemporary restorative concepts are based on four to six implants per jaw, providing adequate biomechanical support under given anatomic conditions typically restricted by bone volume and critical anatomic structures [13,14]. Recent systematic reviews and consensus statements on IFCDs remain inconclusive on the optimal number of implants, consistently emphasizing the need for direct clinical comparative studies [1,8,14,15]. Configurations with six maxillary and four mandibular implants represent the most frequently explored concepts, reflecting a widely accepted compromise between clinical feasibility, anatomical limitations, and patient affordability [1,15,16]. The number and distribution of implants in an IFCD is typically determined considering various anatomic, systemic, treatment-related, and patient-centered factors. These factors comprise, e.g., oral and facial aesthetics, prosthetic design and materials, the timing of occlusal loading, oral hygiene aspects, treatment cost, and patient risk factors [13,17,18]. However, case-individual decisions on the number of implants remain largely empirical [15].

Direct clinical comparisons of four vs. six IFCDs in relatively small patient cohorts have remained inconclusive [19,20]. Differences between four and six IFCDs in terms of implant survival, biological integration, and technical complications have recently been reported by Zhang et al. for subcohorts displaying specific risk factors, i.e., increased age or smoking [21].

To further investigate the role of implant number in immediately loaded IFCDs, this retrospective study compared the implant survival of four vs. six IFCDs using a large dataset (943 patients, 5989 implants) with an extended longitudinal follow-up of up to 17 years. Notably, all patients were treated according to a previously described standardized and systematic treatment and follow-up protocol [17,18]. Of note, the number of implants was systematically derived using a clinical decision support system (CDSS) guided by patient-specific alveolar anatomical parameters, particularly alveolar ridge dimensions. This anatomy-driven treatment approach, embedded within a systematic CDSS, represents a novel consideration for analyzing the impact of implant numbers on the outcomes of immediately loaded IFCDs and distinguishes this dataset from previous studies relying on less standardized and potentially empirically driven treatment delivery [17,18].

## 2. Materials and Methods

### 2.1. Study Setup

This retrospective analysis considered clinical records of patients receiving immediately loaded full-arch treatment following a published standardized anatomy and patient-centered treatment workflow [17,18]. Treatments were provided at one private clinic (Implantology Institute, Lisbon, Portugal) from November 2004 to November 2021. Standardized clinical settings related to patient evaluation, selection and follow-up regimens for examinations on implant status and health, and professional hygiene were reported earlier [18].

This study was conducted according to the guidelines of the Declaration of Helsinki and approved by the Institutional Review Board and Ethics Committee in Lisbon (II2021-05). STROBE (strengthening the reporting of observational studies in epidemiology) guidelines were followed for reporting study data.

### 2.2. Treatment Provision

Implant restorative schemes, comprising either four or six implants per arch, were delivered following a structured clinical decision support system (CDSS). All cases involved full-arch screw-retained fixed complete dentures (IFCDs) with immediate loading protocols. The approach was conceptually similar to All-on-4 or All-on-6 rehabilitations, with adjustments made based on individual anatomic and clinical parameters defined by the CDSS [17,18].

**Figure 1:**
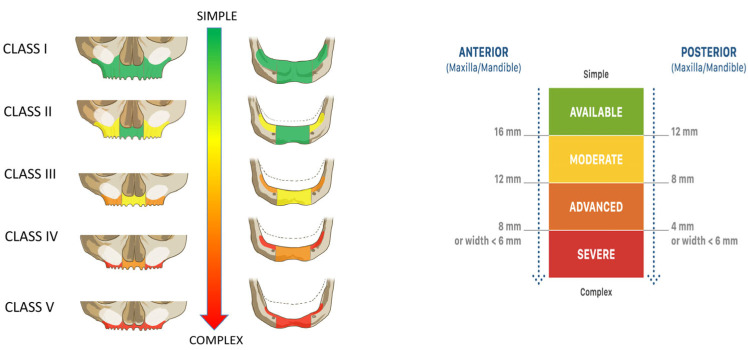
Full-arch anatomical classification (Classes I–V). The classification system categorizes maxillary and mandibular residual ridge conditions based on anterior and posterior alveolar bone height and width. Atrophy severity increases from Class I (adequate volume) to Class V (severely resorbed ridge), with corresponding changes in treatment complexity. The color-coded scale (green to red) reflects the decreasing alveolar dimensions and surgical complexity. Thresholds for the anterior and posterior availability of alveolar bone height and width are defined in millimeters, as shown.

In brief, anatomic criteria comprised the vertical and horizontal alveolar dimensions at three predefined positions per quadrant. These dimensions defined the maxillary and mandibular anatomic classifications (CC), ranging from Class I, indicating no atrophy, to Class V, representing the most severe atrophic condition (Figure 1).

**Figure 2:**
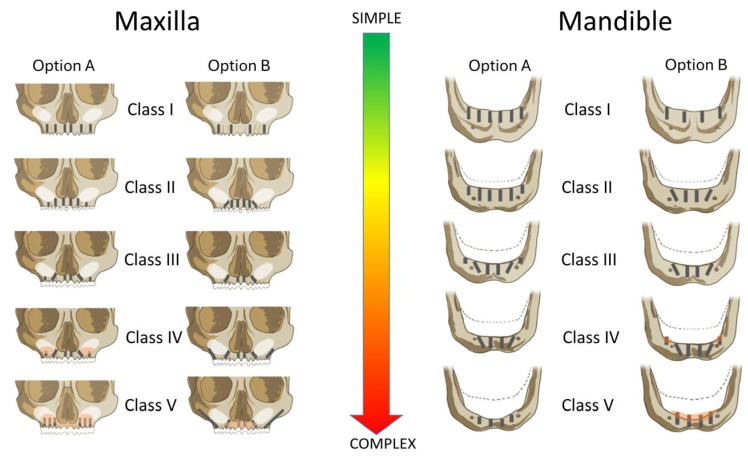
CDSS-guided implant treatment options according to Carames Classification (Classes I–V) for the maxilla and mandible. Option A and Option B represent standardized surgical and restorative strategies tailored to the severity of alveolar atrophy and the presence of risk factors. Treatment complexity increases from Class I (fully preserved ridge) to Class V (severe horizontal and vertical bone loss). The decision tree reflects an anatomical risk stratification approach intended to support predictable full-arch rehabilitation using four or six implants.

Based on the identified class, patients were assigned to one of two predefined implant restorative strategies (Figure 2). These included either four or six immediately loaded implants, with surgical and prosthetic decisions tailored to the anatomical presentation. Furthermore, risk factors like systemic conditions, smoking, and bruxism were considered in the prosthetically driven definition of the implant restorative design, defining the number, type, position, and angulation of the placed implants, use of bone grafting, and the type of prosthetic restoration (Figure 2). The classification thresholds and CDSS-driven workflow were consistently applied across all patients to standardize decision making and reduce inter-operator variability.

Patients exhibiting any contraindication for implant treatment, comprising uncontrolled systemic, local, or psychological conditions, uncontrolled diabetes, pregnancy or nursing, or a history of radiation to the head and neck and patients with a history of intravenous bisphosphonate therapy were excluded from implant treatment. One experienced surgeon (J.M.M.C.) and the clinic’s prosthodontists treated all patients. Implant-restorative treatment concepts and the application of concomitant regenerative therapy followed a standardized workflow based on identical treatment diagnostic CBCT equipment settings (voxel size 0.20 mm, 80 kV, 15 mA, exposure time 12 s, Planmeca Promax, Planmeca, Helsinki, Finland). All implants and materials were used according to the manufacturer’s instructions. Implants were immediately restored and loaded using acrylic provisionals. Porcelain-veneered or monolithic zirconia, ceramic, or acrylic–metal hybrid final prosthesis were delivered 6 months after treatment. Recall regimens were set to a two-weekly interval for the first twelve weeks post surgery and 4-month intervals after that [18].

### 2.3. Dataset-Specific Information

This analysis used a convenience sample identified through a database search, determined by the availability of adequate and complete diagnostic information and follow-up data. The primary dataset was filtered to consider only implant restorations with four or six implants that were entirely delivered within the clinic’s settings and excluding any configurations comprising pre-existing implants. Replacement implants, i.e., implants replacing lost implants and removable prostheses, were not considered.

### 2.4. Outcome and Considered Factor Definitions

The primary outcome of this study was implant survival. For survival analysis, the time to implant failure was defined as the interval between loading and failure based on the presence of clinical or technical signs and symptoms that led to implant removal or placement into sleep [22]. Early and late implant failures were defined as failures before and after 180 days from placement, respectively [23].

The following nominal and categorical factors were considered in the survival analysis:

Implant-placement-related factors included the number of implants per jaw (four vs. six), the jaw type (maxilla, mandible), and placement location as divided into anterior positions (incisors and canine) or posterior positions (pre-molar and molar positions).

Treatment- and material-related factors comprised the implant system by brand and type, implant length and diameter, and the application of concomitant regenerative bone grafting procedures.

Patient-related factors included gender, age at the time of implant placement, self-reported smoking habits, i.e., daily cigarette consumption, and the presence and number of systemic comorbidities, considering cardiac arrhythmia, arthritis, diabetes type I or II, cardiovascular disease, hepatitis B, HIV, arterial hypertension, hyperthyroidism, osteoporosis, and rheumatoid arthritis. Binary predictors categorizing patients into smokers and non-smokers and patients exhibiting potential risk factors were omitted due to multicollinearity with cigarette and comorbidity counts.

The level of alveolar atrophy was classified using a previously described classification [17]. Alveolar atrophy was determined at the quadrant level and classified according to the vertical and horizontal dimensions of available bone at three predefined positions of the alveolar crest. The level of bone atrophy was classified into 5 different maxillary and mandibular categories (CCs), with CC I having the lowest and CC V displaying the highest level of atrophy.

Prosthetic variables, including the type of definitive restoration or restorative material, were not considered in the statistical analysis.

### 2.5. Statistical Analysis

Data analysis was carried out in SPSS for statistical analysis (SPSS software, version 24, SPSS Inc., Chicago, IL, USA) or using RStudio for macOS 2022.07.1+554 (Release 22 July 2022, “Spotted Wakerobin”) and R version 4.2.1. Descriptive characteristics were reported as the means and standard deviations (SDs), medians and interquartile ranges (IQRs), and absolute ranges. The statistical unit for all parameters was the implant. The differences between descriptive values at the study group level were evaluated for statistical significance (*p* < 0.05) using the one-way or Pearson’s Chi-square test or the Mann–Whitney-U test, respectively.

Cumulative survival rates (CSRs) were determined by Kaplan–Meier analysis. Corresponding *p*-values for comparing survival curves and CSRs were calculated using the log-rank test. Hazard risk ratios for the implant loss outcome as a function of the type of implant restoration for the overall and corresponding subcohorts and the analysis of confounding factors were calculated using univariate, i.e., single predictor, Cox proportional hazard models.

A multilevel Cox regression model incorporating a frailty term was built using R’s ‘survival’ package (version 3.3-1) [24]. This frailty term, assuming a Gaussian distribution, accounted for patient-level random effects associated with the placement and longitudinal survival of multiple implants within the same patient. Additionally, it effectively handled unobserved heterogeneity attributable to patient-specific random effects, which could potentially influence implant survival outcomes.

The selection of predictor variables for the model at both the implant and patient levels was guided by causal-directed acyclic graph (DAG) methodologies using the ggdag (version 0.2.10) and dagitty (version 0.3-4) packages in R [25]. From the DAG models delineating all predictors and their hypothetical causal relations presented in Figure S1, two direct open paths between the exposure of interest (prosthesis type) and the outcome (implant survival) were identified (Figure S2).

A minimal adjustment set comprising the predictors' CC class and jaw type (maxilla/mandible) was first defined to estimate the total effect of the prosthesis type on implant survival (Figure S3). Next, a comprehensive adjustment set, referred to as the Canonical Set, was derived to construct a model incorporating a broader range of clinically meaningful predictors beyond the minimal adjustment set [25]. This set included all potential ancestors of the exposure and outcome, excluding any potential descendants of nodes situated on direct causal paths. The Canonical Set comprised the following predictors: CC class, age, tooth location (anterior/posterior), bruxism (yes/no), bone regeneration procedure (yes/no), gender, jaw type (maxilla/mandible), implant length (mm), cigarettes per day, and the number of systemic conditions. Both adjustment sets were examined for potential collider path activation with no indications of collider bias and related selection bias in the models (Figures S4 and S5). Corresponding Cox proportional hazard models using Canonical Sets were employed to analyze the relationship between four vs. six IFCDs and implant loss for the total and the maxillary and mandibular subcohorts. Associations between specific predictor variables and implant loss were considered statistically significant at a *p*-value of ≤0.05.

## 3. Results

The descriptive characteristics for the overall cohort and per maxillary and mandibular subcohorts comprising four or six implants, respectively, are presented in Table 1. This study considered data from 943 treated patients receiving 5989 implants in 2431 quadrants over an average follow-up period of 5.0 ± 3.2 (range 0–17) years. A comparison of the maxillary and mandibular study subcohorts per considered patient/implant characteristic indicated partly significant inter-subcohort differences (Table 1).

### 3.1. Implant Loss and Qualitatively Related Aspects

Four IFCDs did not have a significantly higher relative overall implant loss than six IFCDs, with rates of 1.61% (42 out of 2608 implants) versus 1.21% (41 out of 3381 implants), respectively (*p* = 0.240). The non-significantly higher relative implant loss of four vs. six IFCDs was consistent in both the maxillary (2.4% vs. 1.4%, *p* = 0.130) and mandibular (1.4% vs. 0.6%, *p* = 0.109) subcohorts (Table 1).

Differences in the clustering behavior and the time to implant loss between four and six IFCDs tended to be more pronounced in the mandible than in the maxilla. Specifically, implants in mandibular four-implant restorations failed, on average, after 476.3 ± 713.6 days (67% early losses) and with 26% clustering compared to 111.8 ± 44.8 days (*p* = 0.401) (83% early losses) and 67% clustering (*p* = 0.151) in six-implant restorations. Maxillary implants in four-implant IFCDs were lost on average after 219.5 ± 214.2 days (67% early losses) and with 40% clustering compared to 303.7 ± 295.6 (51% early losses) (*p* = 0.275) and 31% clustering (*p* = 0.379) in six-implant restorations (Table 1).

At the patient level, 68 out of 943 patients (7.2%) experienced at least one implant failure during the follow-up period. In the maxilla, implant loss occurred in 13 of 167 patients (7.8%) treated with four implants and 29 of 427 patients (6.8%) treated with six implants. In the mandible, 24 of 510 patients (4.7%) with four implants and 5 of 180 patients (2.8%) with six implants experienced implant loss. The differences were not statistically significant (*p* = 0.805 and 0.397, respectively)

### 3.2. Kaplan–Meyer Analysis

**Figure 3:**
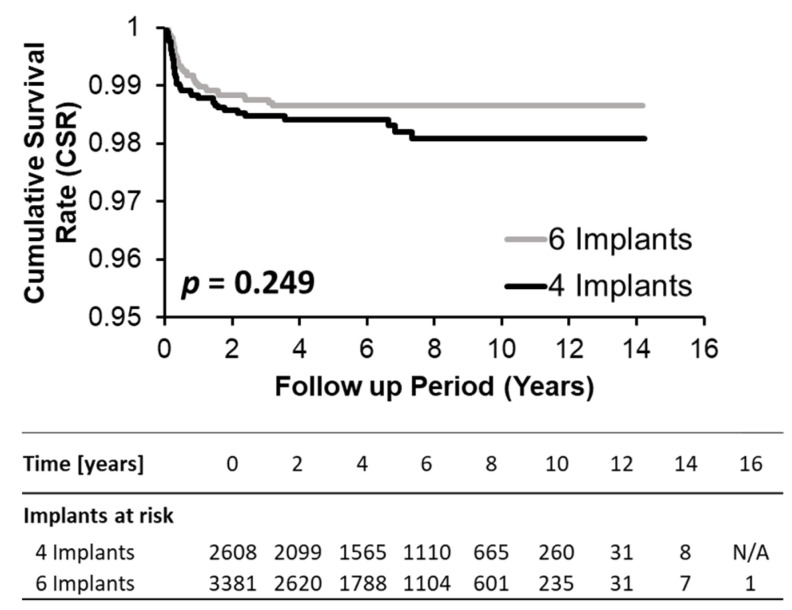
Kaplan–Meier plot comparing cumulative survival rates (CSRs) of four and six implant groups for the overall cohort.

**Figure 4:**
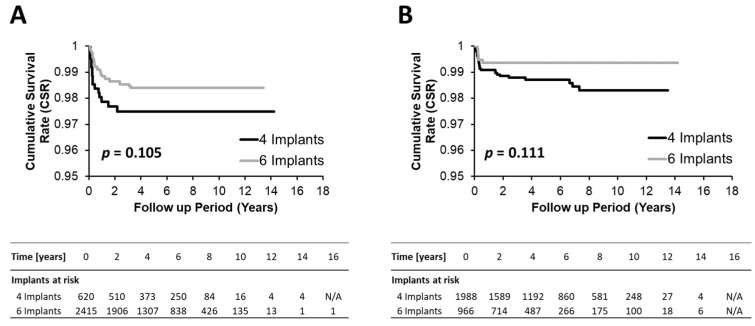
Kaplan–Meyer survival plots comparing the cumulative survival rate of configurations with four and six implants for the maxillary (**A**) and mandibular (**B**) subcohorts. *p*-values were obtained by log-rank tests.

As supported by the Kaplan–Meier curves and corresponding log-rank values in Figure 1 and Figure 2 and the 2- and 5-year cumulative survival rates (CSRs) in Table 2, implant survival in four IFCDs (overall 2-year CSRs 98.6% and 5-year CSR 98.4%) and six IFCDs (overall 2- and 5-year CSRs 98.8% and 98.7%, *p* = 0.362 and 0.343, respectively) were comparable at an overall and maxillary and mandibular level (log-rank *p*-values: overall 0.249, maxillary level: 0.105, mandibular level: 0.111) (Table 2, Figure 3 and Figure 4). Differences between groups were most pronounced in the maxilla and at early time points, remaining below significance (2-year CSR four vs. six maxillary IFCDs: 97.7% vs. 98.6%, *p* = 0.084) (Table 2). Moreover, mandibular survival rates appeared generally higher than maxillary ones (Table 2 and Figure 4).

### 3.3. Single-Predictor Cox Proportional Hazard Ratios

The Cox proportional hazards in Table 3 indicate the comparable risks of implant loss between four and six IFCDs. The same analysis indicated a trend towards significance when comparing maxillary and mandibular equal-implant-number-supported restorations without, however, meeting the conventional thresholds for statistical significance. Specifically, four IFCDs displayed 1.624 (CI 0.886–2.976)- and 1.959 (CI 0.806–4.762)-times-higher risks for implant loss than six IFCDs at the maxillary and mandibular levels, respectively (*p* = 0.117 and 0.138). Similarly, maxillary four and six IFCDs showed a 1.864 (CI 0.990–3.512, *p* = 0.054)- and 2.271 (0.955–5.399, *p* = 0.0635)-times-elevated implant loss risk compared to their mandibular counterparts.

### 3.4. Multilevel Cox Proportional Hazard Models

Multilevel Cox proportional hazard models employing the minimal set adjusting for CC classification and jaw type (maxilla/mandible) resulted in a 1.654-fold (CI 0.913 to 2.999)-increased risk of implant loss for implants in four compared to six IFCDs (Table 4). Differences did not attain the predefined 95% confidence level (*p* = 0.097). The same model revealed a significant 1.950-fold (CI 1.128 to 3.369) higher risk of implant loss in the maxilla compared to the mandible, considering adjustment for restoration type and CC classification (*p* = 0.017).

CC classes showed varying impacts on implant survival when adjusting for restoration and jaw type (Table 4). Specifically, compared to CC1, the hazard ratio was not significant for CC2 (2.112, CI 0.481 to 9.277, *p* = 0.320) and CC3 (2.627, CI 0.593 to 11.641, *p* = 0.200). However, patients classified as CC4 exhibited a 4.420-fold-higher risk of implant failure compared to CC1 without, however, reaching statistical significance (*p* = 0.051). Class CC5 was the only class demonstrating statistical significance, with a 4.951-times-elevated risk for implant loss compared to CC1 (*p* = 0.048).

The clinical, potentially more relevant, multilevel Cox proportional hazard model utilizing the Canonical Set adjusting for a broader range of predictors resulted in a 1.678-fold (CI 0.908 to 3.102)-increased risk of implant loss in four compared to six IFCDs without reaching significance (*p* = 0.098) (Table 5).

Importantly, this model identified a significant 1.815-fold-higher risk (CI 1.037 to 3.175) of implant loss in the maxilla compared to the mandible (*p* = 0.037). Furthermore, only age significantly impacted overall implant survival (HR: 1.026, CI: 1.003 to 1.050, *p* = 0.030). The influence of CC classifications remained below significance for all CCs comprising CC5 (HR: 4.244, CI 0.850 to 21.206, *p* = 0.078). Further, daily cigarette consumption appeared to result in a 1.024-fold (CI: 0.996 to 1.054)-increased risk for implant failure per daily consumed cigarette while remaining below significance (*p* = 0.099). This finding illustrates that both factors increased the hazard ratio by approximately 2% for each additional year or daily consumed cigarette, implying that the difference in age or cigarette consumption between corresponding hypothetical subcohorts should be ten-fold to reach significance. The model’s statistically significant frailty term (*p* = 0.002) indicated substantial unaccounted patient-level heterogeneity.

### 3.5. Multilevel Cox Proportional Hazard Model Analysis at Jaw Level

Cox proportional hazard analysis of the maxillary subcohort indicated a non-significant 1.294-fold (CI 0.327 to 5.122)-increased implant loss risk in four compared to six IFCDs *(p* = 0.710) (Table 6). Age was the only identified predictor for maxillary implant loss with a significant 1.034 (CI 1.001 to 1.066, *p* = 0.038)-times-increased risk per year of age. The statistically significant frailty term suggested considerable unaccounted patient heterogeneity (*p* = 0.003).

Mandibular subcohort analysis also resulted in a non-significant 1.905-fold (CI 0.651 to 5.570)-increased risk for implant loss in four vs. six IFCDs (*p* = 0.240) (Table 7). Bone regeneration and cigarette consumption trended towards an association with implant loss with a 2.211-fold (CI 0.967 to 5.051) (*p* = 0.060)-increased risk of augmented compared to non-augmented sites and a 1.045-fold (CI 0.999 to 1.093) (*p* = 0.054) risk increase per daily consumed cigarette, respectively, without reaching the predefined thresholds for significance.

## 4. Discussion

This retrospective analysis compared implant survival of implant-supported fixed complete dentures (IFCDs) supported by four or six implants. This study provides mid-to-long-term survival data from a large patient cohort, with treatment and restorative approaches being systematically delivered based on a recently published clinical decision support system (CDSS) based on patient, treatment, and anatomical factors [17,18]. In this regard, the presented analysis is the first to systematically document and analyze the impact of implant number and distribution on IFCD survival, considering treatment decisions based on a structured decision-making framework [1,21,27,28].

In summary, this retrospective study identified a range of interesting findings:

- This study indicated high implant survival rates and low implant loss risks irrespective of the number of implants supporting the restoration. Differences in implant survival between four and six IFCDs remained marginal and below statistical significance at overall and jaw-type levels, irrespective of the adopted statistical model.
- Implant survival rates of mandibular implants were higher than maxillary implants, with the jaw type as a risk factor influencing implant loss.
- Four and six IFCDs exhibited qualitative differences in temporal and clustered implant loss profiles, albeit non-significant, warranting further discussion.
- Multilevel regression models identified jaw type and age as confounding factors for implant loss at the global level. The jaw-type level analysis identified age as being associated with maxillary implant loss. Daily cigarette consumption and bone regeneration tended to be associated with implant loss in the mandible.

These findings align with, but also extend, a small body of the literature comparing implant survival in four vs. six IFCDs provided with immediate loading protocols. Indirect comparisons addressing this topic with meta-analytical approaches have remained inconclusive and limited by the relatively high heterogeneity and low comparability between study setups and treatment approaches of the underlying individual studies [1,6,8,13,14,15]. Direct clinical and, specifically, mid- or even long-term direct comparisons, on the other hand, remain, scarce and limited to smaller cohorts. Tallarico et al. recently reported comparable 5-year implant survival outcomes of maxillary four and six IFCDs in a randomized controlled trial in a cohort limited to 40 patients and 200 implants [19]. La Monaca et al., on the other hand, reported a 6.5-year significantly lower CSR of 89.7% for four IFCDs vs. 99.0% for six IFCDs [20]. However, their retrospective studies remained limited by a relatively small patient cohort of 28 subjects and pronounced unexplained clustering of implant loss, respectively. Toia et al. reported comparable 100% and 99% implant survival rates for four to six IFCDs, respectively, in 56 patients with 280 implants over a 3-year follow-up as part of a comparative randomized controlled trial [29].

Only one recent report by Zhang et al. retrospectively compared four and six IFCDs directly in a larger patient cohort of 217 patients with 1222 implants over a 3–13-year follow-up period. While overall survival rates were comparable, Zhang et al. reported significantly lower implant loss hazards for six compared to four IFCDs in specific subcohorts, namely, patients over 60 years of age and moderate to heavy smokers [21]. Notably, these subgroup effects mirror findings in the present study, which also failed to identify significant differences between four and six IFCDs at the overall or jaw-specific levels while confirming age as a significant confounder and identifying cigarette consumption as a potential risk factor, with a non-significant but trending association with implant loss. Differences in treatment provision and, specifically, the application of the CDSS guiding restorative approaches for the herein-reported cohort may limit the comparability between this study and the one reported by Zhang et al.

Biomechanical analyses performed by Skalak and others have illustrated the importance of implant number, location, distribution and orientation in preventing local mechanical implant overload in IFCDs [30,31]. Finite element analysis by Silva et al. could demonstrate that increasing the implant number from four to six reduced the maximum load per implant onto bone [32]. Bevilacqua et al. and Fazi et al. contextualized these findings by demonstrating that distal implant tilting effectively reduced the mechanical overload of distal implants [33,34]. Overall, the current knowledge indicates that the biomechanical advantages of six compared to four implant restorations remain subtle. However, this statement may only be valid considering optimal anatomic preconditions for implant placement, i.e., adequate local bone quantity and quality, i.e., factors that were at least partly considered during treatment provision based on the adopted CDSS [17,18]. To this extent, it is interesting to note that the level of atrophy (CC) was not identified as a robust factor significantly affecting implant loss, supporting the effectiveness of the CDSS to mitigate potential risk factors on implant loss. Likewise, it must be acknowledged that subgroup analysis on the CC level was tempered by small sample sizes and wide confidence intervals.

This analysis confirms previous findings of a significantly higher risk of implant failure in the maxilla compared to the mandible [35,36,37]. This difference has previously been attributed to the maxilla’s lower bone quality and quantity relative to the mandible [36,38]. Specifically, the mandible has been shown to exhibit higher bone density and greater cortical thickness, independent of atrophy levels. In contrast, the posterior maxillary bone is characterized by a thinner cortical bone and a predominantly trabecular architecture, with atrophy levels potentially contributing to reduced bone density [38,39,40]. In this context, it is interesting that Chmielewski et al. recently reported a strong negative correlation between implant stability and patient age, suggesting decreased bone density as a key factor underlying implant loss [41]. Given these considerations, it is notable that the association between atrophy levels and implant loss at the overall level was not robust across statistical models, and no significant association was observed at either the mandibular or maxillary level when the Canonical Adjustment Set was applied. However, the model revealed age to be significantly associated with implant loss in the maxilla but not in the mandible, in contrast to previous observations [27,36]. Thus, while these findings confirm a potential impact of jaw type on implant survival, they also suggest that systematically incorporating atrophy levels into a clinical decision support system (CDSS) may have mitigated the negative effects of alveolar atrophy, reducing its statistical impact [17,18]. Furthermore, these results may also suggest that bone density—although difficult to quantify—represents another important parameter to control implant loss [38,40].

Finally, another interesting observation of this study was that the implant loss profiles of mandibular four IFCDs indicated predominantly early failures as a cause for implant loss. In comparison, six IFCDs showed both early and late failures. However, it must be considered that this trend corroborated with potential, although not statistically significant, differences in clustered implant loss frequencies. Clustered implant failures, as well as the herein identified residual variance in the statistical regression models, may indicate the presence of unaccounted-for patient-level risk factors, e.g., the type and nature of the antagonist, parafunctional habits, and technical complications, which may limit the interpretation of these observations [36].

This study has several limitations that require consideration to interpret the results adequately. These limitations were largely associated with the study’s retrospective design and the intrinsic sample characteristics limitations to capture or control for all potential confounding factors or adequately balance and power these factors among corresponding subcohorts. Additionally, the analysis focused primarily on implant survival without considering the broader context of implant success, including important criteria such as marginal bone loss, implant health-related parameters, or prosthetic outcomes and design. Due to the retrospective design and variability in the availability and standardization of these data across treatment records, such parameters could not be systematically assessed. While current evidence does not consistently support a significant influence of prosthetic material on implant survival in full-arch reconstructions, future studies may benefit from evaluating this factor in more detail. Finally, statistical models revealed the presence of residual patient-level variability not accounted for by the included predictors. Nevertheless, implant survival rates for four and six IFCDs remained consistently high and comparable, regardless of the statistical model or level of analysis.

Future studies may benefit from prospective or randomized trial designs to further evaluate the impact of implant number, prosthetic material properties, and restorative design on both implant and prosthetic outcomes. In addition to survival metrics, broader definitions of implant success—incorporating marginal bone stability, prosthetic complications, and patient-reported outcomes—should be considered. Economic parameters such as treatment cost and long-term cost-effectiveness also warrant further investigation to support patient-centered and evidence-based decision making.

## 5. Conclusions

Four- and six-implant-supported fixed complete dentures (IFCDs), delivered within a systematic patient-centered clinical decision support system (CDSS), demonstrated high and comparable mid-to-long-term implant survival across both overall and jaw-type analyses. This finding remained consistent across all statistical models employed to adjust for confounding factors. Jaw type was a significant predictor of overall implant loss, whereas age was significantly associated with implant loss in the maxilla. In contrast, alveolar atrophy class and related restorative concepts showed no robust association with implant loss, supporting the effectiveness of the adopted patient-centered treatment approach in successfully mitigating the potentially detrimental effects of alveolar atrophy.

## Figures and Tables

**Table 1:** Descriptive characteristics of the total and the maxillary- and mandibular-study-group-specific subcohorts. The statistical unit for all variables was the implant level, except for the patient-level implant loss data in the last row. Descriptive characteristics were compared using one-way or Pearson’s Chi-square test, respectively, or the Mann–Whitney-U test. *p*-values indicating a statistically significant difference between corresponding subcohorts (*p* ≤ 0.05) are marked in bold.

	Total Cohort		Maxilla			Mandible	
Patient/Implant Characteristics		4 Implants	6 Implants	*p*-Value	4 Implants	6 Implants	*p*-Value
Number of restored quadrants	2431	310	805	**<0.001**	994	322	**<0.001**
Number of implants	5989	620	2415	**<0.001**	1988	966	**<0.001**
Average patient age [years]	65.5	65.2 ± 11.9 (35–96)	64.1 ± 10.7 (34–94)	0.204	67.9 ± 11.2 (32–98)	64.7 ± 11.8 (35–96)	**<0.001**
Implants in females vs. males (n/% in females)	3708/2281 (61.9%)	418/202 (67.4%)	1437/978 (59.5%)	**<0.001**	1430/558 (71.9%)	423/543 (43.8%)	**<0.001**
Implants in smokers vs. non-smokers (n/% in smokers)	1487/4502 (24.8%)	164/456 (26.5%)	657/1758 (27.2%)	0.7444	384/1604 (19.3%)	282/684 (29.2%)	**<0.001**
Average daily cigarette consumption among smokers (implant level)	15.7 ± 7.5 (2–40)	18.4 ± 8.8 (2–40)	15.1 ± 7.3 (2–40)	**<0.001**	16 ± 7.5 (2–40)	15.1 ± 6.9 (5–40)	0.0907
Implants in patients with vs. without comorbidities (n/% with comorbidities)	2479/3510 (41.4%)	254/366 (41%)	912/1503 (37.8%)	0.157	926/1062 (46.6%)	387/579 (40.1%)	**0.001**
Average number of comorbidities in patients with ≥1 comorbidity (implant level)	1.2 ± 0.5 (1–5)	1.2 ± 0.5 (1–3)	1.2 ± 0.5 (1–3)	0.771	1.3 ± 0.5 (1–5)	1.2 ± 0.4 (1–3)	**0.0027**
Implants placed with vs. without regenerative procedures (n/% with regeneration)	2309/3680 (38.6%)	224/396 (36.1%)	1343/1072 (55.6%)	**<0.001**	404/1584 (20.3%)	338/628 (5.6%)	**<0.001**
Implants with length ≤8 mm (short implants) (% of total)	5.2%	1.0%	22.0%	**<0.001**	3.0%	12.0%	**<0.001**
Implants with length 8–14 mm (regular implants) (% of total)	15.2%	26.0%	8.0%	**<0.001**	27.0%	3.0%	**<0.001**
Implants with length 15–21 mm (long implants) (% of total)	79.2%	72.0%	86.0%	**<0.001**	70.0%	85.0%	**<0.001**
Implants with length >21 mm (zygoma implants) (% of total)	0.4%	0.20%	0.20%	0.089	0.0%	0.0%	-
Implants lost/not lost (n/% lost)	83/5906 (1.4%)	15/605 (2.4%)	35/2380 (1.4%)	0.130	27/1961 (1.4%)	6/960 (0.6%)	0.109
Early vs. late implant losses (n/% of total losses)	52/31 (62.7%)	10/5 (66.7%)	19/16 (54.3%)	0.871	18/9 (66.7%)	5/1 (83.3%)	0.999
Clustered vs. non-clustered implant losses (n/% of total losses)	24/55 (33.7%)	6/9 (40%)	11/24 (31.4%)	0.379	7/20 (25.9%)	4/2 (66.7%)	0.151
Mean follow-up duration (years, implant level)	5.0 ± 3.2 (0–17)	4.9 ± 2.8 (0.1–14.2)	4.8 ± 3.1 (0–17)	0.322	5.5 ± 3.4 (0–14.7)	4.4 ± 3.3 (0.1–14.2)	**<0.001**
Mean time to implant loss (days)	330.8 ± 472.5 (30–2680)	219.5 ± 214.2 (52–804)	303.7 ± 295.6 (36–1175)	0.275	476.3 ± 713.6 (30–2680)	111.8 ± 44.8 (83–210)	0.401
Patients with ≥1 implant loss (n/% of total patients)	68 (7.2%)	13 (7.8%)	29 (6.8%)	0.805	24 (4.7%)	5 (2.8%)	0.397

**Table 2:** Cumulative implant survival rates (CSRs) after 2 and 5 years of loading as derived from the Kaplan–Meier survival analysis of implants in four and six IFCDs. Results are presented for the total cohort and the maxillary and mandibular subcohorts; *p*-values were derived from log-rank tests and adjusted for multiple comparisons, according to Sidak [26]. Abbreviations: CSR: cumulative survival rate.

	Total Cohort		Maxilla		Mandible	
CSR	4 Implants	6 Implants	4 Implants	6 Implants	4 Implants	6 Implants
2 years	98.6%	98.8%	97.7%	98.6%	98.9%	99.4%
	*p* = 0.362		*p* = 0.084		*p* = 0.224	
5 years	98.4%	98.7%	97.5%	98.4%	98.6%	99.4%
	*p* = 0.343		*p* = 0.105		*p* = 0.136	

**Table 3:** Hazard ratios comparing the risk of four and six IFCDs for implant loss as stratified by the jaw type and between maxillary and mandibular four- and six-implant IFCDs. Risk ratios were calculated from single-predictor Cox proportional hazards models considering the individual as a random effect. Corresponding risk ratios were not explicitly reported. *p*-values indicating a significant difference between tested groups are marked in bold.

Subcohort Level	Risk Factor	Survived Implants/Lost Implant (% lost)	Hazard Ratio (95% CI)	*p*-Value
Maxilla	4	605/15 (2.42%)	1.624 (0.886–2.976)	0.117
	6	2380/35 (1.45%)	1	
Mandible	4	1961/27 (1.36%)	1.959 (0.806–4.762)	0.138
	6	960/6 (0.62%)	1	
4	Maxilla	605/15 (2.42%)	1.864 (0.990–3.512)	0.054
	Mandible	1961/27 (1.36%)	1	
6	Maxilla	2380/35 (1.45%)	2.271 (0.955–5.399)	0.0635
	Mandible	960/6 (0.62%)	1	

**Table 4:** Overall Cox proportional hazard models evaluating the risk for implant loss in the entire cohort after consideration of minimal adjustment set for confounding. The patient was considered in the model as a random effect. Factors with a statistically significant association with implant loss (*p* ≤ 0.05) are marked in bold. Abbreviations: Std Err: standard error; CI: confidence interval.

Outcome	Risk Factor	Value	Hazard Ratio	95% CI	Std Err	*p*-Value
Implant loss	Patient	As a random effect	-	-	-	**0.001**
	Restoration type	4 vs. 6 implants	1.654	(0.913 to 2.999)	0.304	0.097
	Jaw type	Maxilla vs. mandible	1.950	(1.128 to 3.369)	0.279	**0.017**
	Anatomic classification/CC	CC II vs. CC I	2.112	(0.481 to 9.277)	0.755	0.320
		CC III vs. CC I	2.627	(0.593 to 11.641)	0.759	0.200
		CC IV vs. CC I	4.420	(0.994 to 19.650)	0.761	0.051
		CC V vs. CC I	4.951	(1.018 to 24.086)	0.807	**0.048**

Random effect variance = 1.287. Concordance = 0.962 (se = 0.008). Likelihood ratio test = 225.8, df = 94.34, *p* < 0.001.

**Table 5:** Overall Cox proportional hazard models evaluating the risk for implant loss in the entire cohort after consideration of Canonical Adjustment Set for confounding. The patient was considered in the model as a random effect. Factors with a statistically significant association with implant loss (*p* ≤ 0.05) are marked in bold. Abbreviations: Std Err: standard error; CI: confidence interval.

Outcome	Risk Factor	Value	Hazard Ratio	95% CI	Std Err	*p*-Value
Implant loss	Patient	As a random effect	-	-	-	**0.002**
	Restoration type	4 vs. 6 IFCD	1.678	(0.908 to 3.102)	0.313	0.098
	Jaw type	Maxilla vs. mandible	1.815	(1.037 to 3.175)	0.285	0.037
	Anatomic classification/CC	CC II vs. CC I	2.118	(0.478 to 9.374)	0.759	0.320
		CC III vs. CC I	2.727	(0.611 to 12.168)	0.763	0.190
		CC IV vs. CC I	4.452	(0.989 to 20.035)	0.767	0.052
		CC V vs. CC I	4.244	(0.849 to 21.206)	0.821	0.078
	Implantation position	Anterior vs. posterior positions	0.827	(0.518 to 1.321)	0.239	0.430
	Bone regeneration	With vs. without augmentation	1.497	(0.883 to 2.538)	0.269	0.130
	Implant length	Increase per 1 mm	1.043	(0.986 to 1.104)	0.029	0.140
	Age [years]	Increase per 1 year	1.026	(1.003 to 1.050)	0.012	**0.030**
	Gender	Male vs. female	1.120	(0.674 to 1.863)	0.259	0.660
	Bruxism	Present vs. not present	0.971	(0.599 to 1.574)	0.246	0.910
	Cigarettes per day	Increase by 1 cigarette/day	1.024	(0.996 to 1.054)	0.015	0.099
	Number of comorbidities	Increase by 1 comorbidity	1.019	(0.710 to 1.460)	0.015	0.920

Random effect variance = 1.289. Concordance = 0.954 (se = 0.007). Likelihood ratio test = 234.6, df = 99.11, *p* < 0.001.

**Table 6:** Cox proportional hazard models evaluating subcohorts of maxillary implants separately after consideration of Canonical Adjustment Set for confounding. The patient was considered in the model as a random effect. Factors with a statistically significant association with implant loss (*p* ≤ 0.05) are marked in bold. Abbreviations: Std Err: standard error; CI: confidence interval.

Outcome	Risk Factor	Value	Hazard Ratio	95% CI	Std Err	*p*-Value
Implant loss/maxilla	Patient	As a random effect	-	-	-	**0.003**
	Restoration type	4 vs. 6 IFCD	1.294	(0.326 to 5.122)	0.702	0.710
	Anatomic classification/CC *	CC III vs. CC II	1.656	(0.415 to 6.596)	0.705	0.470
		CC IV vs. CC II	2.385	(0.926 to 6.136)	0.482	0.071
		CC V vs. CC II	2.447	(0.900 to 6.650)	0.510	0.079
	Implantation position	Anterior vs. posterior positions	1.019	(0.554 to 1.872)	0.310	0.950
	Bone regeneration	With vs. without augmentation	1.136	(0.566 to 2.280)	0.355	0.720
	Implant length	Increase per 1 mm	1.042	(0.981 to 1.107)	0.030	0.180
	Age [years]	Increase per 1 year	1.033	(1.001 to 1.066)	0.015	**0.038**
	Gender	Male vs. female	1.526	(0.801 to 2.908)	0.328	0.200
	Bruxism	Present vs. not present	1.048	(0.557 to 1.968)	0.321	0.880
	Cigarettes per day	Increase by 1 cigarette/day	1.006	(0.968 to 1.045)	0.017	0.770
	Number of comorbidities	Increase by 1 comorbidity	1.192	(0.746 to 1.905)	0.212	0.460

Random effect variance = 1.444. Concordance = 0.966 (se = 0.006). Likelihood ratio test = 163.8, df = 64.82, *p* < 0.001. * No event (implant failures) was detected for CC I in maxila. Thus, the CC II category has been selected as a reference level.

**Table 7:** Cox proportional hazard models evaluating subcohorts of mandibular implants separately after consideration of Canonical Adjustment Set for confounding. The patient was considered in the model as a random effect. Factors with a statistically significant association with implant loss (*p* ≤ 0.05) are marked in bold. Abbreviations: Std Err: standard error; CI: confidence interval.

Outcome	Risk Factor	Value	Hazard Ratio	95% CI	Std Err	*p*-Value
Implant loss/mandible	Patient	As a random effect	-	-	-	**0.048**
	Restoration type	4 vs. 6 IFCD	1.905	(0.651 to 5.570)	0.547	0.240
	Anatomic classification/CC *	CC II vs. CC I	0.615	(0.114 to 3.315)	0.811	0.570
		CC III vs. CC I	0.813	(0.155 to 4.261)	0.796	0.810
		CC IV vs. CC I	1.239	(0.221 to 6.944)	0.823	0.810
	Implantation position	Anterior vs. posterior positions	0.647	(0.298 to 1.405)	0.395	0.270
	Bone regeneration	With vs. without augmentation	2.211	(0.967 to 5.051)	0.421	0.060
	Implant length	Increase per 1 mm	1.042	(0.981 to 1.107)	0.030	0.180
	Age [years]	Increase per 1 year	1.017	(0.980 to 1.055)	0.186	0.370
	Gender	Male vs. female	0.657	(0.268 to 1.610)	0.456	0.360
	Bruxism	Present vs. not present	0.749	(0.342 to 1.639)	0.399	0.470
	Cigarettes per day	Increase by 1 cigarette/day	1.045	(0.999 to 1.093)	0.022	0.054
	Number of comorbidities	Increase by 1 comorbidity	0.764	(0.420 to 1.389)	0.290	0.380

Random effect variance = 1.444. Concordance = 0.966 (se = 0.006). Likelihood ratio test = 163.8, df = 64.82, *p* < 0.001. * No event (implant failures) in the mandible was detected for CC V.

## Data Availability

The data that support the findings of this study are not publicly available due to data protection regulations.

## Supplementary Materials

The following supporting information can be downloaded at: https://www.mdpi.com/article/10.3390/jcm14124237/s1, Figure S1: Directed Acyclic Graph (DAG) of all predictors and their potential causal connections; Figure S2: DAG illustrating identified direct open pathsl; Figure S3: DAG with minimal adjustment set; Figure S4: DAG showing collider paths (minimal adjustment); Figure S5: DAG showing collider paths (canonical adjustment).
