# Supplementary material for: Four vs. Six Implant Full-Arch Restorations—A Direct Comparative Retrospective Analysis in a Large Controlled Treatment Cohort
Source: J Clin Med. 2025 Jun 14;14(12):4237. doi: 10.3390/jcm14124237 (PMC12194759; doi:10.3390/jcm14124237)

Survival of Implants Supporting Full-Arch Immediate Fixed Prostheses with Four vs. Six Implants: A Causal Inference-Based Retrospective Cohort Study

João Manuel Mendez Caramês [1,2,3,*], Helena Cristina Oliveira Francisco [1,2,] Filipe Araújo Vieira [1,2], Gonçalo Bártolo Caramês [1], Jorge Nuno do Rosário Martins[1,2] and Duarte Nuno da Silva Marques [1,2,3]

[1] Instituto de Implantologia, Avenida Columbano Bordalo Pinheiro, nº50, 1070-064 Lisbon, Portugal; filipevieira@edu.ulisboa.pt.vieira@gmail.com (F.A.V.); caramesgoncalo@gmail.com (G.B.C.); jnr_martins@yahoo.com.br (J.N.R.M); helenafrancisco@campus.ul.pt (H.C.O.F.); duarte.marques@campus.ul.pt (D.N.d.S.M.)
[2] Faculdade de Medicina Dentária, Universidade de Lisboa, 1600-277 Lisbon, Portugal
[3] LIBPhys-FCT UID/FIS/04559/2013, Faculty of Dental Medicine, University of Lisbon, 1600-277 Lisbon, Portugal
* Correspondence: carames@campus.ul.pt; Tel.: +351-91972-7353; Fax: +351-21721-0989

Supplementary Information

Figure S1: *Directed Acyclic Graph (DAG) of all predictors and their potential causal connections.*

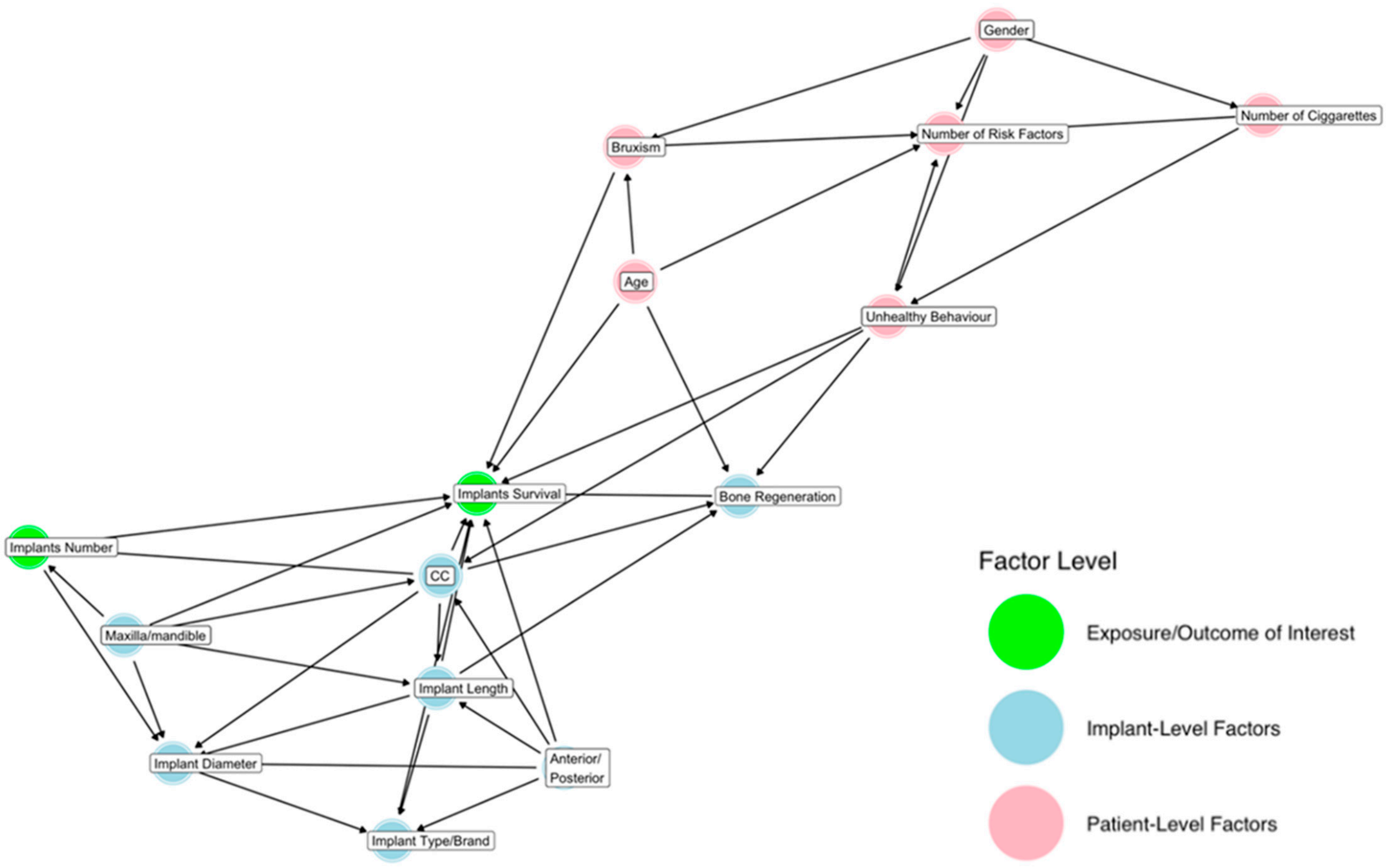

Figure S2*: Directed Acyclic Graph model illustrating the identified direct open paths as potential causal paths between the number of implants per prosthesis and implant survival.*

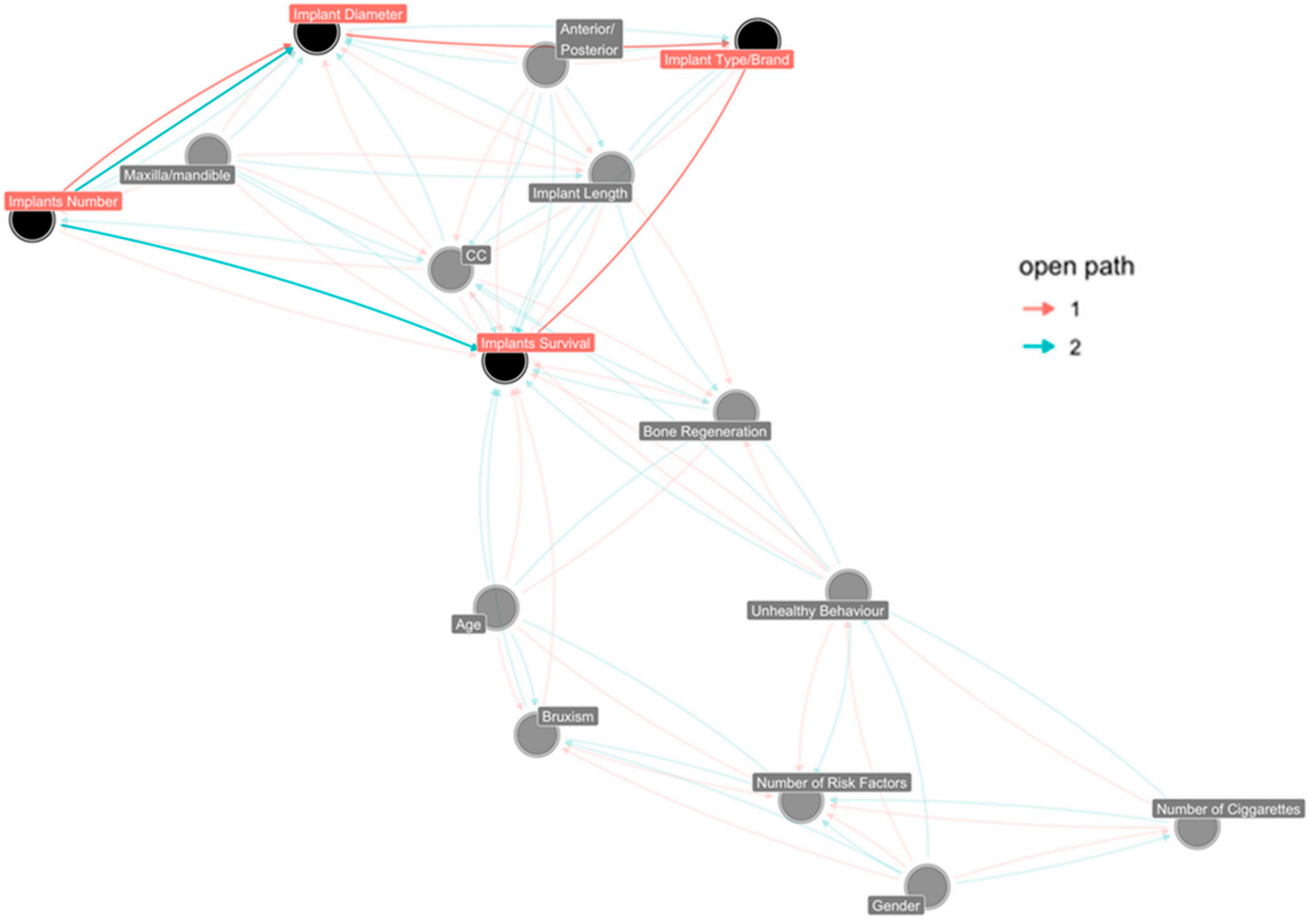

Figure S3: *Directed acyclic graph model considering a minimal adjustment set as the smallest set of confounding variables used to estimate the total effect of prosthesis type on implant survival.*

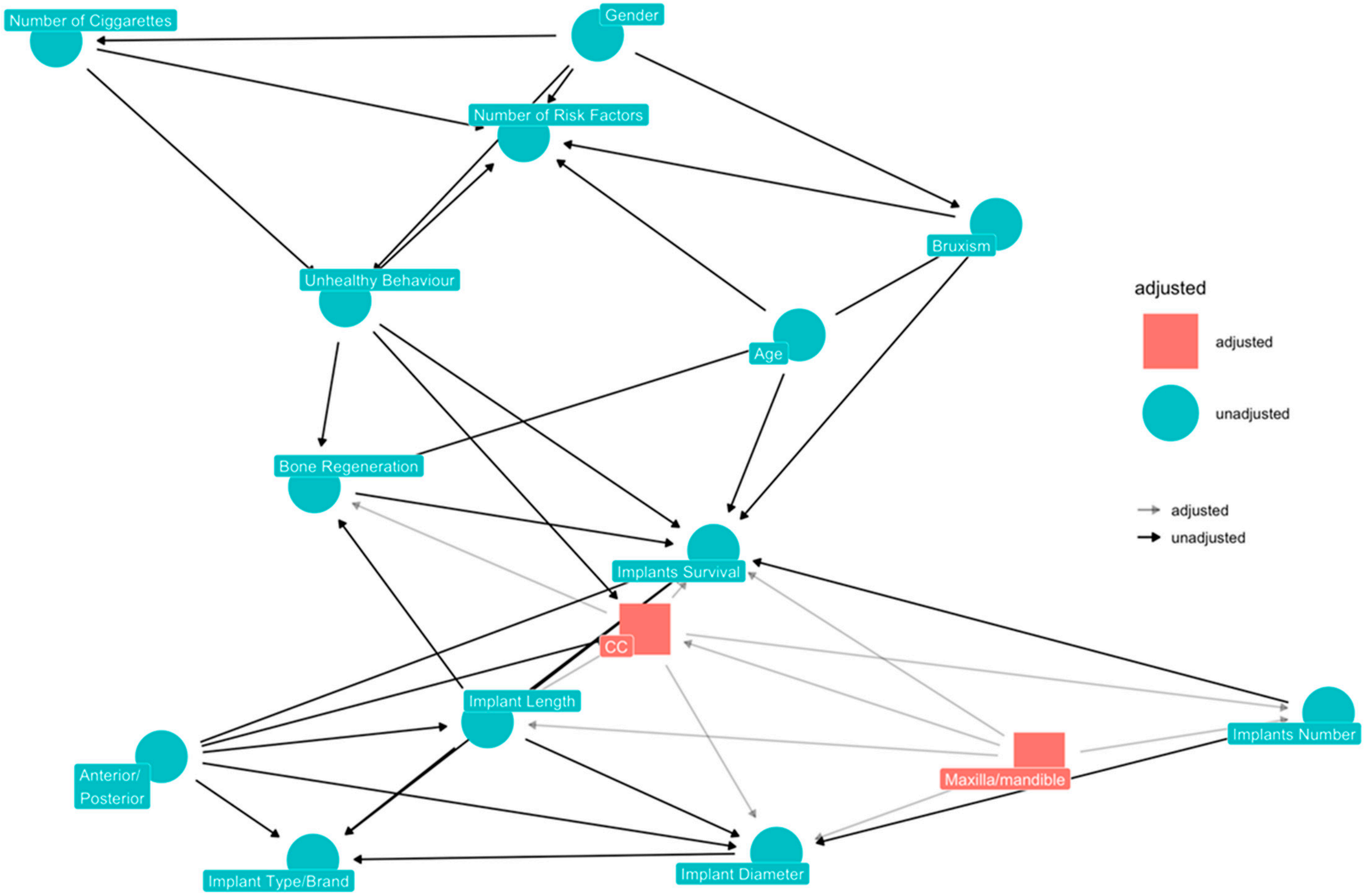

Figure S4: *Directed acyclic graph model illustrating the collider paths assessment performed for the minimal adjustment set. No new activated collider paths between Implants Number and Implants Survival were identified.*

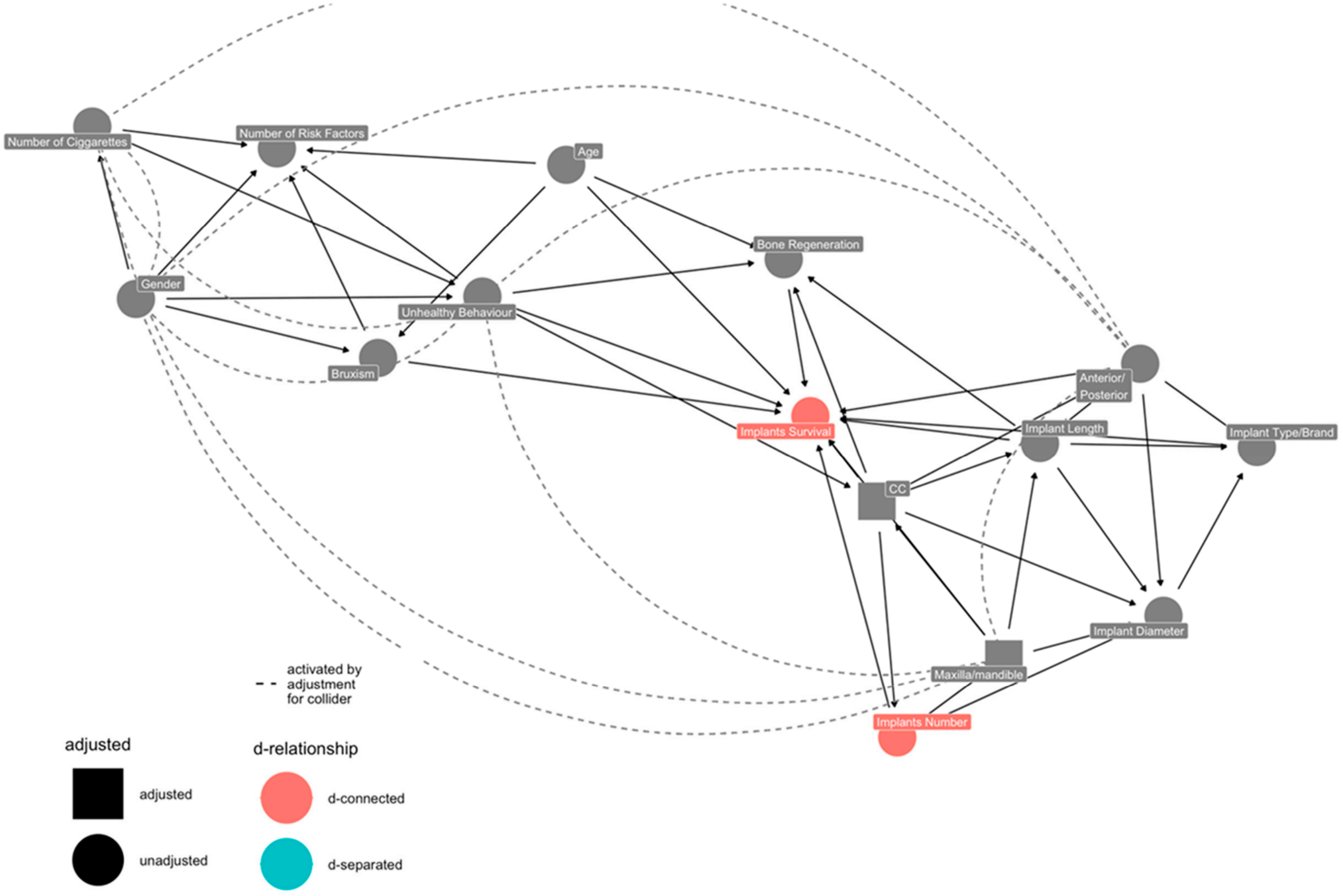

Figure S5: *Directed acyclic graph model illustrating the collider paths assessment performed for the canonical adjustment set. No new activated collider paths between Implants Number and Implants Survival were identified.*

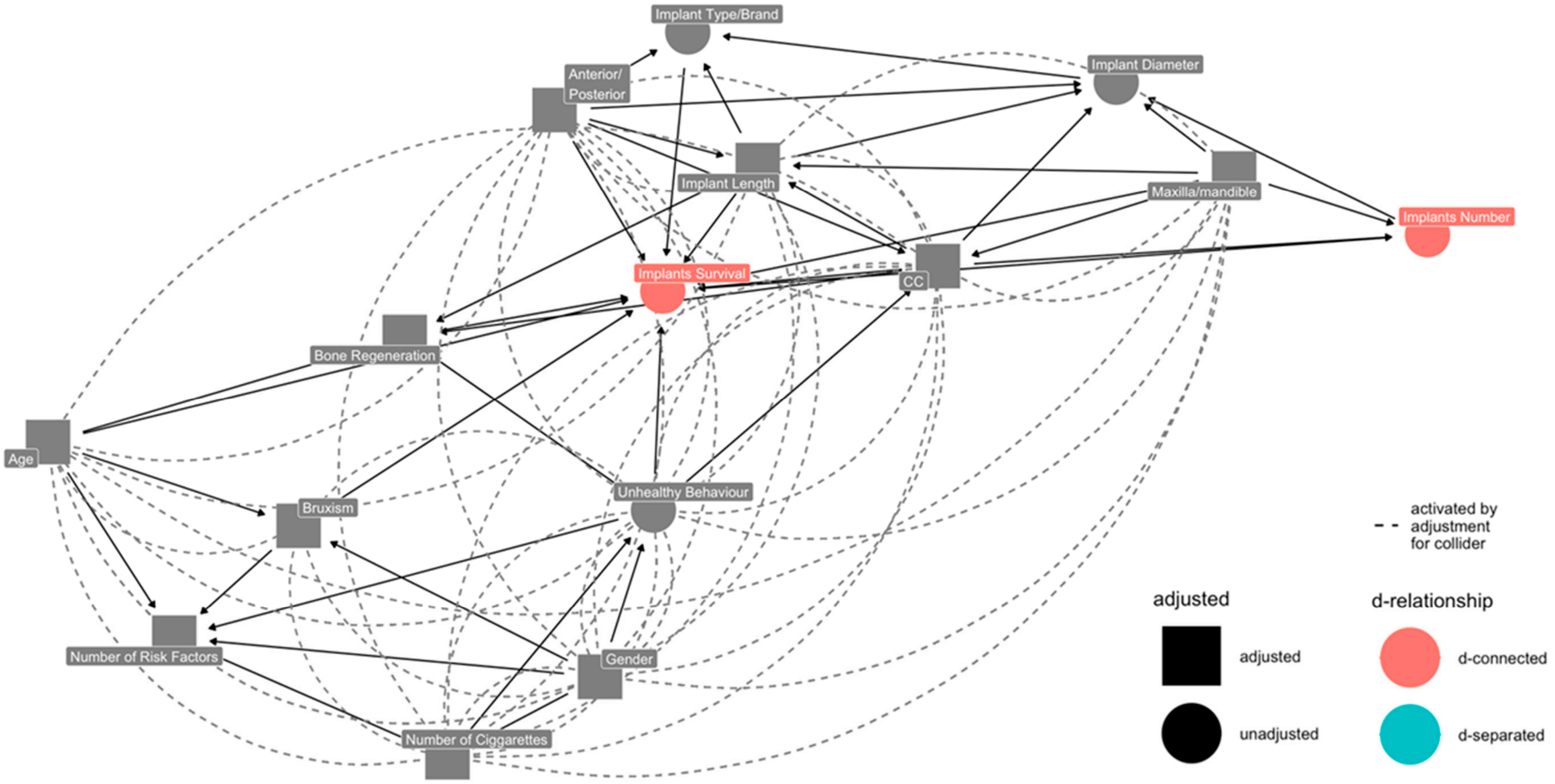

Supplement: Supplementary file 1 [file jcm-14-04237-s001.zip › jcm-3631170-supplementary.pdf]
